# From Parasite to Mutualist: Rapid Evolution of *Wolbachia* in Natural Populations of *Drosophila*

**DOI:** 10.1371/journal.pbio.0050114

**Published:** 2007-04-17

**Authors:** Andrew R Weeks, Michael Turelli, William R Harcombe, K. Tracy Reynolds, Ary A Hoffmann

**Affiliations:** 1 Department of Genetics, Centre for Environmental Stress and Adaptation Research, University of Melbourne, Parkville, Victoria, Australia; 2 Section of Evolution and Ecology and Center for Population Biology, University of California Davis, Davis, California, United States of America; 3 Section of Integrative Biology, University of Texas, Austin, Texas, United States of America; University of Lausanne, Switzerland

## Abstract

*Wolbachia* are maternally inherited bacteria that commonly spread through host populations by causing cytoplasmic incompatibility, often expressed as reduced egg hatch when uninfected females mate with infected males. Infected females are frequently less fecund as a consequence of *Wolbachia* infection. However, theory predicts that because of maternal transmission, these “parasites” will tend to evolve towards a more mutualistic association with their hosts. Drosophila simulans in California provided the classic case of a *Wolbachia* infection spreading in nature. Cytoplasmic incompatibility allowed the infection to spread through individual populations within a few years and from southern to northern California (more than 700 km) within a decade, despite reducing the fecundity of infected females by 15%–20% under laboratory conditions. Here we show that the *Wolbachia* in California D. simulans have changed over the last 20 y so that infected females now exhibit an average 10% fecundity advantage over uninfected females in the laboratory. Our data suggest smaller but qualitatively similar changes in relative fecundity in nature and demonstrate that fecundity-increasing *Wolbachia* variants are currently polymorphic in natural populations.

## Author Summary

*Wolbachia* are endosymbiotic bacteria that live inside the cells of their invertebrate hosts. They are transmitted directly from mother to offspring, and spread through populations by manipulating the reproduction of their hosts. The most common reproductive manipulation responsible for the spread of these bacteria, called “cytoplasmic incompatibility,” arises when infected males mate with uninfected females, resulting in fewer offspring than normal. There are fitness costs for the hosts associated with *Wolbachia* infections, most commonly involving a reduction in egg production. Theory predicts that this detrimental effect of *Wolbachia* on its host should result in selection for the bacteria to evolve a more benign lifestyle, changing the bacterium from being parasitic to more mutualistic. We document such a shift in a *Wolbachia* infection of fruit flies *(Drosophila simulans)* from California. The shift occurred extremely rapidly, over 20 years. Consequently, *Wolbachia*-infected hosts now have higher rates of egg production than their uninfected counterparts. Changes in the genome of *Wolbachia* seem to be responsible for this, rather than changes in the host genome. Our study reveals that bacteria and their hosts represent components of a dynamic interacting system that can evolve rapidly over time.

## Abstract 3

A parasitic endosymbiont,*Wolbachia,* has rapidly evolved towards a more mutualistic lifestyle with its*Drosophila* host in under 20 years, suggesting a dynamic interaction between parasitic and mutualistic lifestyles.

## Introduction

When microbes that live within animal cells are transmitted only maternally, their reproductive success is directly tied to that of the matrilines they inhabit. Both intuition and mathematics suggest that such endosymbionts will be selected towards mutualism, if possible, increasing the fecundity of their female hosts [1]. The expectation that vertical transmission favours evolution towards mutualism is supported by both laboratory co-evolution experiments between viruses and bacteria and comparative data from a wide range of natural associations [1,2]. Mutualisms generally have long evolutionary histories, but given the potentially explosive rate of bacterial evolution [3], rapid evolution of mutualisms in nature might also be expected. Here we report such evolution by bacteria *(Wolbachia)* associated with a dipteran host *(Drosophila simulans)* in natural California populations. In less than 20 y, the *Wolbachia* in California D. simulans have changed so that infected females now produce more eggs than uninfected females under laboratory conditions, whereas infected females previously suffered a significant fecundity deficit.

Cytoplasmic incompatibility (CI) in insects is normally caused when *Wolbachia*-infected males mate with uninfected females or females that carry a different *Wolbachia* strain [4]. Because CI causes embryo lethality, infected females, who are protected from CI, often have a reproductive advantage over uninfected females. This results in the rapid spread of the infection through host populations when the *Wolbachia* are faithfully transmitted from mother to offspring and produce relatively minor fitness costs.

Hoffmann et al. [5] discovered *Wolbachia*-induced CI in California populations of D. simulans. Initially, the California *D. simulans Wolbachia* (*w*Ri) infection was found only south of the Tehachapi transverse range in the southern quarter of the state. From 1985 to 1994, we monitored the infection's spread north [6,7]. We showed that the dynamics and equilibrium infection frequencies in nature could be described accurately by a discrete-generation model with only three parameters: μ, the average frequency of uninfected ova produced by an infected female; *H,* the relative hatch rate from “incompatible” fertilisations of uninfected eggs by sperm from infected males (the other three possible fertilisations produce indistinguishable hatch rates); and *F,* the relative fecundity of infected females [8–10]. Using replicated field assays in the early 1990s, we found the following: μ ≈ 0.045, *H* ≈ 0.55, and *F* ≈ 1.0. In contrast, in laboratory populations, the infection showed perfect maternal transmission (μ = 0), and infected females were 10%–20% less fecund than uninfected females (*F* ≈ 0.8–0.9). Our field-based parameter estimates produce a predicted equilibrium infection frequency, *p̂* ≈ 0.94, consistent with data from several locations, including three populations where we monitored the infection's introduction and spread over about 2 y, with dynamics that roughly matched our simple predictions [7]. The *w*Ri infection quickly spread northward through California and is now pervasive throughout most North American populations of D. simulans.

Models suggest that both *Wolbachia* infections and the host nuclear background should evolve to reduce deleterious effects associated with the infection and to increase the transmission fidelity of the microbe [11,12]. Despite the fact that CI allows *Wolbachia* to spread within populations, intrapopulation selection of *Wolbachia* is not expected to directly affect the level of CI [11,12], unless host populations are structured so that kin selection favours more intense CI [13] (e.g., when infected males can reduce the larval competition experienced by the progeny of their female siblings, a condition that is likely to be rare for D. simulans). In contrast, host evolution is expected to reduce the intensity of CI (i.e., increase embryo viability from incompatible crosses) [12]. Both *Wolbachia* and host evolution affecting transmission (μ), level of incompatibility *(H),* and fecundity of infected hosts *(F)* are plausible, because both *Wolbachia*- and host-related effects influence CI, transmission, and fitness [14–16]. Indeed, some *Wolbachia* infections do not induce detectable levels of CI and have no known deleterious effects on host fitness [17,18]. Positive fitness effects from *Wolbachia* infections have been suggested [19–22], and indirect comparative evidence from different *Wolbachia* infections in *Drosophila* indicates that *Wolbachia–*host interactions may become more benign and potentially mutualistic over time [4,23].

Southern California populations of D. simulans are natural candidates for observing such evolution, because they have been stably infected for at least two decades, the host populations produce on the order of 10–15 generations per year [7], and the parameter values describing transmission, fecundity, and CI level are such that both the infection and its host should experience significant pressure to evolve [12]. We have informally monitored these populations for evolutionary changes in CI and other *Wolbachia* effects for about a decade. Here we provide our accumulated evidence that the *Wolbachia* infection in D. simulans has changed to become more mutualistic, while no evolution by either the *Wolbachia* or D. simulans has been detected affecting CI levels or the fidelity of maternal transmission.

## Results

### Fecundity Effects in the Laboratory

**Figure 1:**
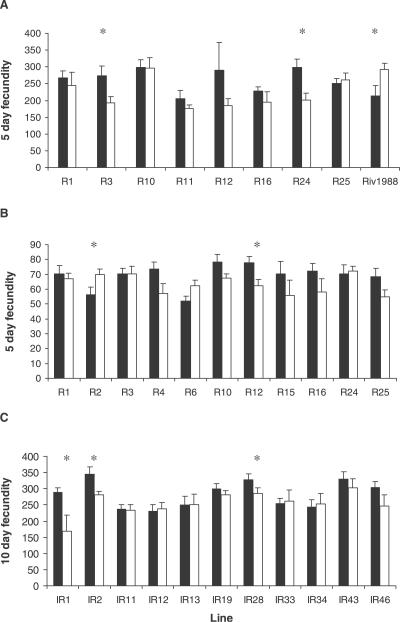
Fecundity Assays on Infected and Uninfected Isofemale Lines of D. simulans Collected from California in 2002 and 2004 (A) Mean number of eggs over 5 d laid by infected (black bars) and uninfected (white bars) lines collected at Riverside in 2002 and 1988. The 2002 lines were assayed four generations after collection. A significant overall increase in fecundity was associated with the infection (*F*_1,108_ = 6.7, *p* = 0.011). (B) Mean number of eggs over 5 d laid by infected (black) and uninfected (white) lines collected at Riverside in 2002 and assayed 10 mo after collection. These flies were cultured on a yeast-restricted diet. Again, the infection produced a significant overall increase in fecundity (*F*_1,213_ = 5.8, *p* = 0.017). (C) Mean number of eggs over 10 d laid by infected (black) and uninfected (white) lines collected at Irvine in 2004 and assayed five generations after collection. Infection status had a significant effect on fecundity (*F*_1,185_ = 8.03, *p* = 0.005). Error bars are standard errors, and asterisks indicate significant differences at the 5% level.

Apart from the viability effects associated with CI, female fecundity is the only fitness component known to be affected by the *w*Ri infection in California D. simulans [9]. To test whether fecundity effects associated with this *Wolbachia* infection have evolved, we re-examined female fecundity in California D. simulans after 20 y [5]. In the late 1980s, fecundity costs were evident in the laboratory when lines treated with the antibiotic tetracycline, which cures *Wolbachia* infections, were compared to untreated lines, or when naturally uninfected and infected lines were compared [9]. Flies were collected in 2002 and uninfected lines were generated by treatment with tetracycline. Infected and uninfected lines were then scored for fecundity every day over 5 d. Overall, there was a fecundity advantage associated with the infection; in some lines no fecundity advantage was detected, while in other lines the total egg output was significantly greater in infected individuals (Figure 1A). In contrast, the *w*Ri line collected from Riverside in 1988 and maintained in the laboratory still produced a fecundity deficit, comparable to the deficits found in infected lines previously (see Table 10 of [8] and Table 7 of [9]). We repeated this fecundity assay, again curing many of the same lines after 10 mo of laboratory culture. To test the sensitivity of the fecundity effect to culture conditions, we restricted access to yeast. Overall, the infected lines still showed greater fecundity than the uninfected lines derived from them. Although one line showed a decrease in fecundity when infected, there was a significant increase overall, and no significant interaction effect (Figure 1B). When individual lines were considered, there appeared to be a shift in fecundity for line R3 and R24 (Figure 1A and 1B). However, when we computed confidence intervals on these data for the difference in fecundity relative to the uninfected lines, the bootstrapped confidence intervals (bCIs) overlapped between, for example, the yeasted R3 line (mean difference 42%, 95% bCI 3.7% to 85%) and non-yeasted R3 line assays (mean difference 0.4%, 95% bCI −6.8% to 20.4%). Hence, the lack of repeatability for the statistical significance of individual lines is likely to reflect primarily the large inherent stochasticity of fecundity data. In contrast, the positive effect of the *Wolbachia* infection on mean fecundity across lines is evident in both of the experimental treatments reported in Figure 1A and 1B (the mean effect is 23% in Figure 1A and 10% in Figure 1B).

Because *Wolbachia* effects on fitness may be temporarily or permanently induced by tetracycline treatment [14,24], we re-collected isofemale lines of D. simulans from Irvine, California, in 2004. We then generated uninfected lines from these and reciprocally crossed infected and uninfected sublines two generations after tetracycline treatment (using old males in the incompatible cross) to homogenise nuclear backgrounds and remove the effects of the antibiotic treatment. Fecundity was then assayed each day over 10 d, controlling for body size. Again, a significant overall fecundity advantage of approximately 10% was associated with *Wolbachia* infection (*F*_1,185_ = 8.03, *p* = 0.005), with three lines out of eleven (IR1, IR2, and IR28) showing a significant fecundity advantage (Figure 1C). Therefore, the fecundity effect associated with the *w*Ri infection in the laboratory has changed from negative to positive. The apparent lack of consistency of this fecundity advantage across individual lines suggests that there may be polymorphism among the *Wolbachia* strains infecting the populations near Riverside and Irvine. These data do not demonstrate polymorphism because there is no significant line-by-infection interaction in our fecundity assays. However, polymorphism is expected from our theoretical analyses (see “Mathematical Analyses”) and is directly demonstrated from additional data presented below.

Our data indicate that the fecundity deficit initially associated with the *w*Ri infection in laboratory assays [7,9] has disappeared. We computed 95% CIs of the mean difference between infected and uninfected lines following Sokal and Rohlf (p. 444 of [25]) for the 2004 Irvine data and earlier published data (Table 10 of [9]; Table 7 of [7]). The mean difference for infected minus uninfected lines from the 2004 data was 10.4% (95% bCI 2.6% to 18.2%) and for the earlier data was −19.4% (95% bCI −24.5% to −13.6%). Thus, the infection has changed from causing a significant fecundity deficit to causing a significant fecundity advantage in the laboratory (an overall shift of 30%).

### Fecundity Effects

#### *Wolbachia* or *Drosophila* evolution?

**Figure 2:**
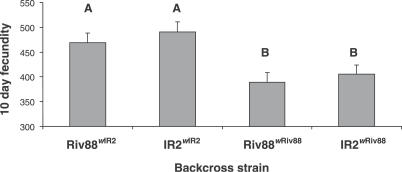
Effects of *Wolbachia* Strain and Nuclear Background on 10-d Fecundity of D. simulans The nuclear background (normal text) and *Wolbachia* strain (superscript text) are for flies collected in Riverside in 1988 (Riv88) or Irvine in 2004 (IR2). Error bars are standard errors. Different letters indicate significantly different means (at the 5% level).

We have shown that the fecundity deficit associated with the *w*Ri infection has changed into a fecundity benefit in less than 20 y, while (as shown below) both CI and transmission levels have stayed roughly constant. But what is causing this effect? To determine whether the fecundity benefit is caused by the *Wolbachia* or an interaction between *Wolbachia* and its host, we backcrossed for five generations the old *w*Ri strain collected in 1988 into the IR2 (Irvine isofemale line 2; Figure 1C) nuclear background, and the IR2 *Wolbachia* strain into the Riverside 1988 nuclear background. We then assayed fecundity as before over 10 d and found a clear strain effect (*F*_1,77_ = 18.3, *p* < 0.001) but no effect of nuclear background (*F*_1,77_ = 0.95, *p* = 0.33) or interaction between nuclear background and *Wolbachia* strain (*F*_1,74_ = 0.02, *p* = 0.88) (Figure 2). This supports the previous finding in our fecundity assays that showed no interaction of line (nuclear genome) by infection (*F*_10,175_ = 1.06, *p* = 0.40). Thus, the observed change in the fecundity effect of *Wolbachia* infection appears mostly due to *Wolbachia* evolution.

#### *Wolbachia* polymorphism?

**Figure 3:**
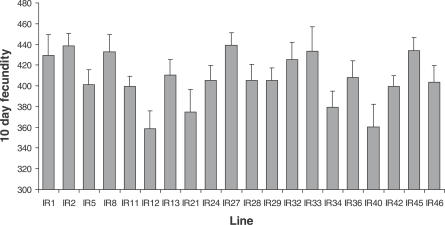
Effects of *Wolbachia* Strain on 10-d Fecundity of D. simulans Collected at Irvine in 2004 and Backcrossed for Two Generations to Males from Riverside Collected in 1988 Error bars are standard errors.

To test whether the *Wolbachia* advantage is polymorphic in the Irvine 2004 collection, we backcrossed for two generations the nuclear background of the old *w*Ri line collected in 1988 into 20 strains from the Irvine 2004 collection so that we could determine strain effects on a similar nuclear background (at least 75% homogeneous). Fecundity was assayed as before over 10 d, and we found a clear strain effect on fecundity (*F*_19,290_ = 2.49, *p* < 0.001) (Figure 3). Some residual nuclear effects are likely to contribute to this strain difference, but polymorphic *Wolbachia* effects are also likely to be involved, consistent with the variable differences between cured and infected lines described above. Coefficients of variation calculated from the data for Figure 1C were larger (15% for infected lines and 12% for uninfected lines) than for the data in Figure 3 (6%) when nuclear effects were controlled.

### CI Levels

**Figure 4:**
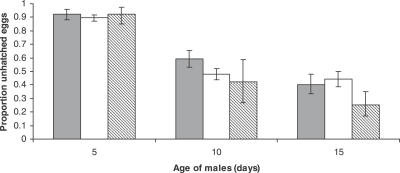
Levels of CI Induced by *Wolbachia*-Infected D. simulans Males 5, 10, and 15 d Post-Eclosion When Mated to Uninfected Females Isofemale lines originated from California in 1988 (hatched bars), 1999 (grey bars), and 2002 (white bars). Error bars are 95% confidence intervals.

Evolutionary changes may also influence CI levels and maternal transmission. To test for changes in CI, we reciprocally mated infected and uninfected individuals derived from isofemale lines of D. simulans collected in 1999 and 2002 and compared these with the old *w*Ri line collected at Riverside in 1988. We also mated females to males that were 5, 10, and 15 d post-eclosion, as male age can affect CI [7,9,26]. When males were 5 d old, levels of CI were high, with an average of 92% egg mortality for all lines in crosses between uninfected females and infected males (Figure 4). CI levels did drop off when males were 10 and 15 d old, as described previously [7,9]. However, there was no difference in the level of CI between the Riverside collections in 1999 and 2002 and the old 1988 *w*Ri line, and the power of our CI tests indicated that we could have detected a difference of 8% or greater. Hence, the level of CI has certainly evolved significantly less than the fecundity effects observed in our laboratory assays.

The levels of CI for matings involving males in the three age classes are also similar to those found previously [7,9]. We assume that the lack of change of CI in our laboratory assays implies relative constancy of CI levels in nature. Hence, we expect that *H,* the average relative hatch rate from an incompatible fertilisation in nature (sperm from an infected male fertilising an uninfected ovum), remains approximately 0.55, as estimated previously [7].

### Maternal Transmission and Population Frequencies

We did not directly re-measure field maternal transmission rates of the *Wolbachia* infection. Instead, we used field infection frequencies and our assumption that *H* remains approximately 0.55 to indirectly estimate transmission efficiency, as any significant increase or decrease in maternal *Wolbachia* transmission would lead to an observable change in the equilibrium infection frequency. For instance, with *H* = 0.55, explaining an equilibrium frequency of 0.94 requires a transmission efficiency of 0.964 to 0.953 if the relative fecundity of infected females, *F,* is between 0.9 and 1.1. Our previous estimates of transmission frequency in nature, denoted 1 − μ, averaged 0.96, consistent with the observed infection frequency and field estimates of CI intensity and relative fecundity [7]. Because selection among mutually compatible *Wolbachia* variants acts to increase the parameter combination *F*(1 − μ) [12], we expect μ to decrease. If the failure rate of maternal transmission, μ, was halved to 0.02 (which would have roughly the same fitness impact on *Wolbachia* as increasing *F* by only 2%), the expected equilibrium infection frequencies would rise to 0.97, with *H* = 0.55 and *F* between 1.0 and 1.1 (see “Mathematical Analyses”).

We collected 654 D. simulans females from four locations in California (Irvine, Ivanhoe, Riverside, and Winters), and screened F_1_ females from each field-collected female for *Wolbachia* using PCR. We found that infection frequencies did not differ significantly between the locations, with an overall infection frequency of 93% (Table 1; *G* test for homogeneity, *G* = 3.09, df = 3, *p* = 0.38). These infection frequencies do not differ from those found in earlier studies [7,9], suggesting that transmission of *Wolbachia* from mother to offspring (1 − μ) has not changed appreciably.

**Table 1:**
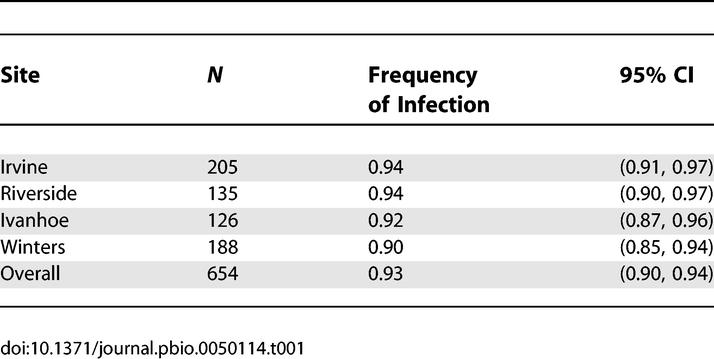
*Wolbachia* Infection Frequency in Four Populations of D. simulans from California in 2004

### In Situ *Wolbachia* Evolution or Invasion of a New Strain?

To determine if the observed fecundity advantage of *w*Ri infection is associated with evolution of the *w*Ri strain and not merely a new strain of *Wolbachia* that has invaded California, we tested for compatibility between the old *w*Ri line collected in 1988 and the IR2 line collected from Irvine in 2004. If *Wolbachia* strains are incompatible (i.e., cause CI in matings between lines), then it is likely that a new strain has invaded California populations of D. simulans. However, we found no difference between the hatch rates of crosses between and within lines (*F*_3,72_ = 0.08, *p* = 0.97), indicating complete compatibility between the *Wolbachia* strains. In addition, we sequenced part of the Wolbachia wsp gene (611 bp) for the old *w*Ri strain collected in 1988 and strains from 25 isofemale lines collected at Irvine in 2004 and found no differences at the nucleotide level. We also sequenced part of the *Wolbachia ftsZ* gene (718 bp) for five of these strains and found no differences in nucleotide sequence compared to the 1988 *w*Ri strain. Therefore, it is likely that the change in fecundity has involved evolution of the *w*Ri strain rather than invasion by a new strain.

### Fecundity Effects in Nature versus the Laboratory

Essentially all studies of *Wolbachia* effects have been limited to laboratory populations. The *w*Ri infection of D. simulans is one of very few whose effects have been studied in nature (cf. [27–29]). We have shown that *w*Ri has apparently evolved in nature over the past 15 y so that laboratory isofemale lines with the infection tend to show a fecundity advantage on the order of 10% rather than a fecundity disadvantage on the order of 20%, as was the case when the *w*Ri infection was new in California D. simulans. We do not yet have new fecundity data from wild-collected females to directly compare fecundities of infected versus uninfected females in nature.

However, we previously reported such data while we tracked the northward spread of *w*Ri through California. During this spread, we were able to study populations in which *w*Ri was at intermediate frequencies, allowing us to make comparisons between the fecundities of wild-collected infected versus uninfected females. We performed nine comparisons, four in the Tehachapi mountains of southern California in 1988–1989 (Table 4 of [9]), then five more near Davis in northern California in 1992–1993 (Tables 6 and 7 of [7]) (all of these data are summarised in Table S1). Collectively these studies assayed the fecundity of more than 1,000 females from nature, and each of our nine comparisons was based on 45–203 females. Only the first of these nine studies (104 females) found a statistically significant fecundity deficit for infected females (*F* = 0.82, *p* < 0.05). More relevant to our new laboratory fecundity data is that the four 1988–1989 studies produced an average relative fecundity of *F* = 0.92, whereas the five 1992–1993 studies produced an average of *F* = 1.03 (with three of the last five point estimates for *F* above one). Although the difference between these two sets of estimates is marginally non-significant in a one-tailed test (*t*-test, *t* = 1.76, df = 7, *p* = 0.06), it suggests that the changes in relative fecundity we have documented in the laboratory reflect similar changes in nature. This is discussed further below in light of our theoretical analyses.

## Discussion

In less than 20 y, the *w*Ri strain that invaded and spread throughout California in the 1980s has evolved from inducing a fecundity deficit in the laboratory to providing a fecundity benefit to its host, as theory predicts [12]. There has been no detectable change in the level of CI, indicating that the genes controlling fecundity have at most minor pleiotropic effects on CI. Rapid evolutionary change within this system has resulted in a parasitic *Wolbachia* evolving towards a more mutualistic interaction with its host. Interspecific comparisons (e.g., [30–32]) and laboratory experimental evolution systems (e.g., [33]) provide many examples supporting the theoretical prediction that vertical transmission, as opposed to horizontal transmission or a mixed mode of transmission, tends to promote mutualism [34–36]. There are well-known examples in which viruses have rapidly evolved to become more benign in nature [37,38]. However, these are best interpreted as evolution towards an “optimal” level of virulence, rather than evolution towards mutualism [34]. We know of no previous examples in which an evolutionary shift towards mutualism has been observed over a period of decades in nature.

To understand the evolutionary dynamics in nature that have so rapidly produced the new fecundity effects, we assume—consistent with our laboratory CI data—that the relevant *Wolbachia* variants are fully compatible with each other. This implies that within the population of infected individuals, the frequencies of alternative *Wolbachia* types follow haploid selection dynamics with fitness determined by the parameter combination *F*(1 − μ), irrespective of whether the variants cause different levels of CI with uninfected individuals [12]. Between 1988 and 2002, the California populations of D. simulans have produced about 200 generations [7]. The observed fecundity variation produced by different *Wolbachia* on a common genetic background (Figure 3) demonstrates polymorphism for the fecundity-increasing *Wolbachia* variant(s). Irrespective of within-host dynamics, we can use discrete-generation haploid selection theory to explore the selective pressures responsible for the spread of fecundity-enhancing variants among hosts and their likely evolutionary trajectory (see “Mathematical Analyses”).

Assuming that the observed changes are attributable to increased frequency of variants initially present, but extremely rare, in the 1988 southern California *w*Ri population, our analysis suggests that selective advantages in the field are likely to be on the order of 5% (whereas 1% or 15% are unlikely). Theory also indicates that the current polymorphism should be transient and that the fecundity-enhancing variants should reach very high frequencies in these populations over the next 5–10 y. Hence, we predict that the continuing evolution of these *Wolbachia* populations will be easily documented.

Our data on compatibility of the “new” versus “old” *Wolbachia* variants and their DNA sequence similarity indicate that *Wolbachia* effects on its host evolve readily in natural populations by selection among closely related *Wolbachia* variants. Such rapid evolution helps to explain the diversity of effects of *Wolbachia* on host fitness noted in the literature: these effects range from negative [9,15,39] to positive [19,21,22] to the extreme where *Wolbachia* becomes essential for host survival [20] or host fertility [40,41]. It also helps to explain the inconsistent effects of *Wolbachia* on host fitness detected in previous experiments [22,42]; changes in the apparent host effects of *Wolbachia* over time or between experiments may well reflect selection among *Wolbachia* variants rather than residual effects of antibiotics or changes in *Wolbachia* density. The rapid evolution of *w*Ri, as well as rapid evolution of *Wolbachia* hosts [43], suggests a dynamic interaction between parasitic and mutualistic life modes and rapidly changing effects of endosymbionts in host insect evolution.

## Materials and Methods

### Strains.

The CI assays used D. simulans collected from Riverside, California, in 1998 and 2002 and maintained in the lab as isofemale lines until testing. Fecundity assays included the 2002 isofemale lines and those established from females collected at Irvine, California, in 2004. A California *w*Ri-infected line from 1988 was included in some assays. To determine the infection frequencies in California populations, approximately 200 female D. simulans were collected at each of four localities (Irvine, Ivanhoe, Riverside, and Winters) in 2004, and F_1_ individuals scored for infection status by PCR assay (described below).

To produce uninfected sublines for each line, larvae were treated with 0.03% tetracycline [5] for two generations. Lines were reared for at least two generations without tetracycline before the CI and fecundity experiments.

### CI assay.

Level of CI was determined by mating virgin 5-, 10-, and 15-d-old *Wolbachia*-infected males to uninfected virgin females (>5 d old) from the same 1998 and 2002 collected lines. Reciprocal crosses acted as controls. Males were mated once, and females were placed after mating in a vial with a spoon containing 5 ml of agar-treacle-yeast medium and left for 24 h at 25 °C. The number of unhatched eggs was counted >24 h later.

CI data (egg hatch rates) were angular transformed prior to analysis. Model I ANOVA (analysis of variance) and *t*-tests were used to compare CI levels between the Riverside collections from 1998 and 2002 and the *w*Ri line from 1988.

### Fecundity assay.

Five fecundity experiments were done. In the first two (Figure 1A and 1B), lines from the 2002 Riverside collection were cured, and infected and uninfected females from each line were mated to uninfected males from the same line. In the first experiment, the 1988 *w*Ri line was included to re-test the previously described fecundity deficit [9]. Flies were reared at low densities by placing 20 eggs per vial on 15 ml of medium. To measure fecundity of emerging flies, pairs of 1-d-old virgin females and males were placed in vials with spoons as for the CI tests. Spoons were replaced every 24 h for 5 d and eggs counted. Between ten and 15 females were assayed for each line. Yeast paste was added to the medium surface in the first experiment, but not in the second experiment, to see if the same fecundity-enhancing *Wolbachia* effect could be detected when egg output was suppressed due to the absence of live yeast.

In the third experiment (Figure 1C), lines from the 2004 Irvine collection were cured as above. To control for nuclear background, we crossed uninfected and infected flies from the same line reciprocally and scored F_1_ offspring for fecundity (with live yeast) after they had been reared and set up as above. Fecundity scoring was extended from 5 to 10 d to increase the likelihood of detecting small differences. Between 15 and 20 replicates were assayed per infected/uninfected treatment of each isofemale line. Wing size (measured as centroid size based on landmarks [44]) was also measured for each female and used as a covariate in analyses to control for body size.

To assign the effects on fecundity to either *Wolbachia* or a host–*Wolbachia* interaction, we backcrossed the nuclear background of one Irvine line showing the greatest fecundity advantage (Figure 1C; IR2) into the 1988 *w*Ri strain, and the 1988 *w*Ri line nuclear background into the IR2 strain, both for five generations (Figure 2). Ten-day fecundity was measured on 20 replicate pairs of males and females per backcross line as above. *Wolbachia* strain and nuclear background were treated as fixed effects in the ANOVA for fecundity.

Finally, to determine whether the *Wolbachia* fecundity effect was polymorphic within the 2004 Irvine lines, we backcrossed the 1988 *w*Ri line nuclear background into 20 strains (isofemale lines) from the 2004 Irvine collection for two generations (Figure 3). Ten-day fecundity was measured on 20 replicate pairs of males and females as above. Model I ANOVA was used to determine *Wolbachia* strain differences for fecundity. We also determined the coefficient of variation [25] for the lines in this experiment and the infected and uninfected lines from the third fecundity experiment (2004 Irvine lines) to see if they fitted the patterns expected (fecundity of infected > fecundity of uninfected > fecundity of infected with a homogenised background).

### *Wolbachia* infection status.

We determined the infection status of all lines collected from the field or after treatment with tetracycline using extracted DNA from females in a PCR with the *Wolbachia*-specific primers 76–99 forward and 1012–994 reverse which amplify a ~ 950-bp fragment of bacterial 16S rDNA [45]. The D. melanogaster primers su(s) forward 724–753 and su(s) reverse 1113–1092 were included in each reaction as a control [7].

To determine the infection frequency in the four populations collected from California in 2004, we first assayed DNA as above from a single F_1_ female from each field-collected female. In addition, another PCR with the *Wolbachia*-specific primers *wsp*81F and *wsp*619R [46] was performed with the same DNA to minimise the chance of false positives. If either or both of these assays were negative, DNA was extracted from a second F_1_ fly from the same isofemale line and subjected to the two PCRs. This second fly was used to control for PCR artefacts and imperfect maternal transmission of *Wolbachia* [7,9].

### CI data comparing the compatibility of old versus new infections.

To determine compatibility between the 1988 *w*Ri line and the IR2 line collected at Irvine in 2004, we crossed virgin males and females (>5 d old) between and within each line in a reciprocal design. Males were mated once, and females were placed after mating in a vial with a spoon containing medium as above for the CI assays. The number of unhatched eggs was counted after 48 h. The analysis was as above for the CI assays.

### Sequence data comparing the similarity of old versus new infections.

To compare the similarity between the 1988 *w*Ri strain and the Irvine strains collected in 2004, we sequenced 611 bp of the highly variable Wolbachia wsp cell-surface gene [46] and 718 bp of the *Wolbachia ftsZ* cell-cycle gene. DNA was extracted from a single female from each of 25 isofemale lines from the 2004 Irvine collection and the laboratory line of the 1988 *w*Ri strain. The partial *wsp* gene fragment was amplified from all lines using the primers and protocol found in [46]; the partial *ftsZ* gene was amplified from only five isofemale lines from the 2004 Irvine collection and the 1988 *w*Ri line as in [47]. Amplified fragments were sequenced using the BigDye Terminator cycle sequencing kit (v3.1, Applied Biosystems, http://www.appliedbiosystems.com). Sequences were aligned using the CLUSTAL W algorithm [48]. We also included in the analysis the original *wsp* and *ftsZ* sequences of the *w*Ri strain found in GenBank (http://www.ncbi.nlm.nih.gov/Genbank; accession numbers AF020070 and U28178, respectively).

### Mathematical analyses.

We analysed various mathematical models to address three issues discussed in the text: (1) inferences concerning transmission-rate evolution based on the dependence of equilibrium infection frequencies on the three parameters that are sufficient to explain dynamics and equilibria in nature [7], (2) the intensity of selection responsible for the observed evolution, and (3) predicted future frequency changes in the fecundity-enhancing *Wolbachia* variant(s). Our methods and analyses leading to our conclusions are described below.

Regarding dependence of equilibria on parameter values, to make inferences concerning whether *Wolbachia*'s maternal transmission rate has evolved, we first considered how the stable equilibrium infection frequency, denoted *p̂*, changes with the parameters *H,* the relative hatch rate from incompatible crosses, *F,* the relative fecundity of infected versus uninfected females, and μ, the fraction of uninfected ova produced by an infected female. Based on field estimates of infection frequencies, we concluded that the equilibrium frequency throughout central and southern California in 1992 was approximately *p̂* ≈ 0.94 (with 95% confidence interval 0.92 to 0.96) [7]. This was consistent with our theoretical prediction for *p̂* from field-based parameter estimates [7]. Our new laboratory data suggest that *F* has evolved significantly, while *H* has remained relatively constant. Given the change in *F,* it may seem surprising that our new estimate of the infection frequency in central and southern California, approximately 93% (with 95% confidence interval 0.90 to 0.94), does not differ significantly from the frequency estimated previously. Our formula for *p̂* allows us to examine the consistency of these observations.

Evolutionary theory suggests that if *Wolbachia* variants remain fully compatible, *F*(1 − μ) should tend to increase [12]. Thus, we are particularly interested in determining whether μ has decreased. However, because fitness is proportional to *F*(1 − μ), changing *F* from 0.9 to 1.0 or from 1.0 to 1.1 involves a selection coefficient on the order of *s* = 0.1 (which should produce significant changes in polymorphic *Wolbachia* variant frequencies over tens of generations). In contrast, halving μ from 0.04 to 0.02 involves much weaker selection, on the order of *s* = 0.02, so that hundreds of generations would be required for significant evolution. Hence, we expect that detectable evolutionary changes since the mid-1980s in μ are much less likely than detectable changes in *F*.

**Figure 5:**
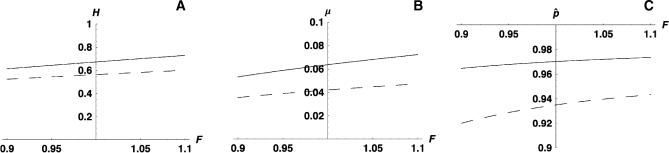
Effects of Changes in Parameters for Various Values of *F* (A) Assuming μ = 0.04, the solid line shows the value of *H* needed to produce *p̂* = 0.90, and the dashed line shows the value of *H* needed to produce *p̂* = 0.94. (B) Assuming *H* = 0.55, the solid line shows the value of μ needed to produce *p̂* = 0.90, and the dashed line shows the value of μ needed to produce *p̂* = 0.94. (C) Assuming *H* = 0.55 and μ = 0.045 (dashed line) or μ = 0.0225 (solid line), this shows how *p̂* changes.

Figure 5A and 5B explore how varying *F* changes the values of *H* and μ necessary to explain equilibrium population frequencies of 0.90 or 0.94. Figure 5 assumes μ = 0.04 and shows the values of *H* needed to produce *p̂* = 0.90 (solid line) versus 0.94 (dashed line) as *F* varies from 0.9 to 1.1. As shown, varying *F* over this range requires very little change in *H* to preserve *p̂*. Similarly, Figure 5B assumes *H* = 0.55 and shows the values of μ needed to produce *p̂* = 0.90 (solid line) versus 0.94 (dashed line) as *F* varies from 0.9 to 1.1. Again, changing *F* has little effect. Both graphs indicate that changes in *F* are likely to have little impact on *p̂*. This is shown directly in Figure 5C, which assumes *H* = 0.55 and μ = 0.045 (or μ = 0.0225) and plots *p̂* as *F* varies from 0.9 to 1.1. Clearly, changes in *F* over the range suggested by our laboratory and field data have little impact on *p̂*. In contrast, a change in μ that would have a much smaller impact on *Wolbachia* fitness would produce changes in *p̂* that our samples would have detected.

Regarding selection intensity, within the population of infected individuals, the frequencies of mutually compatible *Wolbachia* variants follow haploid selection dynamics with the fitness of each variant proportional to *F*(1 − μ), irrespective of the level of CI they produce with uninfected females [12]. All else being equal, two conclusions follow: (1) the level of CI is not subject to direct selection based on between-host frequency dynamics, and (2) for values of *F* near one and μ near zero (as suggested by our data), selection for modifying *F* is much stronger than selection for modifying μ. These inferences are consistent with our data, which suggest that *H* and μ have remained relatively constant, while *F* has increased.

To make quantitative inferences, we assumed discrete generations. If we consider two *Wolbachia* variants such that *F*_1_(1 − μ_1_)/[*F*_2_(1 − μ_2_)] = 1 + *s,* the frequency of variant 1 in generation *t,* denoted *p_t_,* changes according to

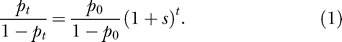



Our inferences about plausible selection intensities follow from equation 1, assuming that the observed changes have occurred over roughly 200 generations.

**Figure 6:**
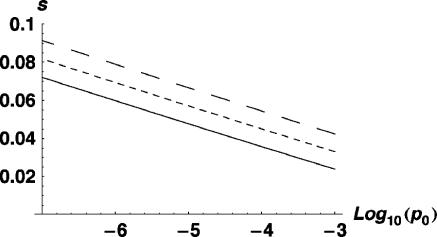
Selection Intensity Needed to Explain a Transient Polymorphism Intensity of selection, *s,* needed to explain a current frequency of 0.8 (solid line), 0.4 (short-dashed line), or 0.1 (long-dashed line) as a function of the initial allele frequency measured in units of log_10_, assuming equation 1.

**Figure 7:**
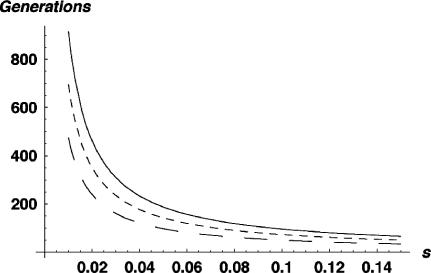
Time Required for a Favoured Variant to Spread Number of generations required for a favoured variant to go from an initial frequency of 0.001 to a final frequency of 0.1 (long-dashed line), 0.5 (short-dashed line), or 0.9 (solid line) as a function of the intensity of selection, *s,* assuming equation 1.

Figure 6 illustrates the selection intensity needed to explain a transient polymorphism, assuming that the fecundity-enhancing variant was rare in the population 200 generations ago. It shows that for low initial frequencies (say, between 10^−3^ and 10^−6^), selection coefficients, *s,* on the order of 0.04–0.08 would produce polymorphic frequencies (between 0.1 and 0.8, for instance) that are consistent with our data. In contrast, for *s* on the order of 0.01, a significantly longer time would be necessary to produce the polymorphism observed (see Figure 7), whereas if *s* were as large as 0.15, polymorphism for the fecundity-enhancing variant would tend to be very short-lived (on the order of 2 y).

Regarding future evolution, our analysis suggests that the currently inferred polymorphism for the fecundity-enhancing variant(s) is likely to be transient. We can use equation 1 to understand the time scale over which near-fixation is expected. Figure 7 plots the time required for a favoured variant to increase from an initial frequency of 0.001 up to a frequency of between 0.1 and 0.9. The difference between the highest and lowest lines indicates the time required for the frequency to increase from 0.1 to 0.9. Note that for *s* as large as 0.15, this time is only about 31 generations, or approximately 2 y in these populations. Given that samples collected in 2002 and 2004 both showed an apparent polymorphism, it seems unlikely that selection was this intense. This inference is consistent with our conjecture that the current *Wolbachia*-induced fecundity advantage in nature is likely to be less than the 10% effect observed in the laboratory, just as the fecundity deficit of roughly 15% found in the laboratory in 1989 [9] corresponded to a fecundity deficit for infected field-collected females that was probably less than 10% in 1989 [9] and less than 8% in 1992 [7]. Conversely, if the fecundity advantage was as small as 1% (corresponding to *s* = 0.01), as Figure 7 shows, the inferred polymorphism would be unlikely to arise in only 15 y (about 200 generations). Hence, for plausible levels of selection, we are likely to be able to observe significant frequency increases of the fecundity-enhancing *Wolbachia* variant(s) in nature over the next few years.

## Supporting Information

Table S1 Previously Published Relative Fecundities, *F,* of Wild-Caught Infected versus Uninfected Females (31 KB DOC)

### Accession Numbers

Sequences for the *wsp* and *ftsZ* genes sequenced in this study have been deposited in GenBank (http://www.ncbi.nlm.nih.gov/Genbank) under the accession numbers EF423730–EF423735 for *ftsZ* and EF423736–EF423761 for *wsp*.

## Abbreviations

bCI: bootstrapped confidence interval
CI: cytoplasmic incompatibility
*w*Ri: *Drosophila simulans Wolbachia*
