# Supplementary material for: From Parasite to Mutualist: Rapid Evolution of Wolbachia in Natural Populations of Drosophila
Source: PLoS Biol. 2007 Apr 17;5(5):e114. doi: 10.1371/journal.pbio.0050114 (PMC1852586; doi:10.1371/journal.pbio.0050114)
Supplement: Supplementary file 1 [file pbio.0050114.st001.doc]

**Supporting Information**

**Table S1. Previously published relative fecundities, *F*, of wild-caught infected versus uninfected females.**

| Population | Month/Year of Collection | *N* | *F* |
| --- | --- | --- | --- |
| Tehachapis | 7/1988 | 104 | 0.82 |
|  | 7/1988 | 52 | 0.99 |
|  | 11/1988 | 173 | 0.97 |
|  | 8/1989 | 75 | 0.90 |
|  |  |  |  |
| Davis/Winters | 11/1992 | 45 | 0.92 |
|  | 11/1992 | 164 | 1.08 |
|  | 3/1993 | 95 | 0.94 |
|  | 3/1993 | 105 | 1.04 |
|  | 4/1993 | 203 | 1.06 |
